# Mytoxin B and Myrothecine A Induce Apoptosis in Human Hepatocarcinoma Cell Line SMMC-7721 via PI3K/Akt Signaling Pathway

**DOI:** 10.3390/molecules24122291

**Published:** 2019-06-20

**Authors:** Huiliang Song, Yi Fu, Dan Wan, Wenjing Xia, Fengwei Lyu, Lijun Liu, Li Shen

**Affiliations:** 1 Institute of Translational Medicine, Medical College, Yangzhou University, Yangzhou 225001, China; 18362825104@163.com (H.S.); 18252717215@163.com (D.W.); wenjingxiajj@163.com (W.X.); yzdxyxyyx@163.com (F.L.); 2 Jiangsu Key Laboratory of Integrated Traditional Chinese and Western Medicine for Prevention and Treatment of Senile Diseases, Yangzhou University, Yangzhou 225001, China; 3 School of Biology and Basic Medical Sciences, Soochow University, Suzhou 215123, China; yfu@suda.edu.cn; 4 Jiangsu Co-Innovation Center for Modern Production Technology of Grain Crops/Jiangsu Key Laboratory of Crop Genetics and Physiology, Yangzhou University, Yangzhou 225009, China; ljliu@yzu.edu.cn; 5 Jiangsu Co-Innovation Center for the Prevention and Control of Important Animal Infectious Diseases and Zoonoses, College of Veterinary Medicine, Yangzhou University, Yangzhou 225009, China

**Keywords:** trichothecene macrolide, SMMC-7721 cells, anticancer mechanism, apoptosis, PI3K/Akt signaling pathway

## Abstract

Trichothecene macrolides comprise a class of valuable leading compounds in developing anticancer drugs, however, there are few reports concerning their anticancer mechanisms, especially the anticancer mechanism of the 10,13-cyclotrichothecane derivatives that are found mainly in symbiotic fungi. In vitro anticancer activity of two trichothecene macrolides mytoxin B and myrothecine A against the human hepatocarcinoma cell line SMMC-7721 was investigated in the present study. MTT assay showed that mytoxin B and myrothecine A inhibited the proliferation of SMMC-7721 cells in dose- and time-dependent manners. Annexin V-FITC/PI dual staining assay revealed that mytoxin B and myrothecine A both could induce SMMC-7721 cells apoptosis in a dose-dependent manner. The decreased expression level of anti-apoptotic protein Bcl-2 and the increased expression level of pro-apoptotic protein Bax were observed apparently in Western blot analysis. The reduced ratio of Bcl-2/Bax further confirmed the apoptosis-inducing effect of mytoxin B and myrothecine A on SMMC-7721 cells. Moreover, the expression levels of caspases-3, -8, and -9, and cleaved caspases-3, -8, and -9 were all upregulated in both mytoxin B and myrothecine A-treated cells in Western blot analysis, which indicated that both compounds might induce SMMC-7721 cells apoptosis through not only the death receptor pathway but also the mitochondrial pathway. Finally, mytoxin B and myrothecine A were found to reduce the activity of PI3K/Akt signaling pathway that was similar to the effect of LY294002 (a potent and specific PI3K inhibitor), suggesting that both mytoxin B and myrothecine A might induce SMMC-7721 cells apoptosis via PI3K/Akt pathway.

## 1. Introduction

**Figure 1:**
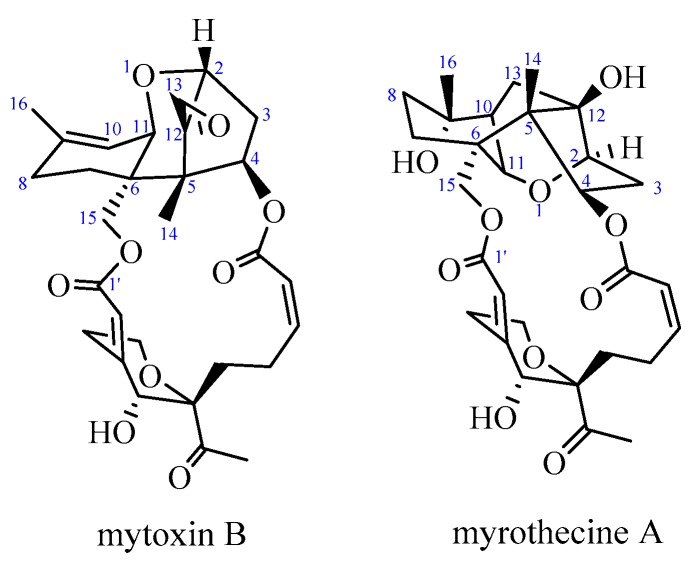
Structures of mytoxin B and myrothecine A.

Biotoxin is an important resource for developing new anticancer drugs and some biotoxins have been successfully used in clinical therapy, such as vincristine and camptothecin. As one kind of mycotoxin, trichothecene macrolides have attracted a lot of attention in the development of anticancer drugs. Trichothecene macrolides, structurally with an additional ring between C-4 and C-15 attached at the 12,13-epoxy trichothecene skeleton, are usually produced by various species of *Myrothecium* sp. [1], *Stachybotrys* sp. [2], *Verticimonosporium* sp. [3], *Calcarisporium* sp. [4], and *Cylindrocarpon* sp. [5]. Trichothecene macrolides displayed significant in vitro and in vivo cytotoxic activity against tumor cells [6,7]. The simple trichothecene, anguidine, had been tested in a phase II clinical study for advanced breast cancer [8], central nervous system tumors [9], and sarcomas unresponsive to prior chemotherapy [10], amongst others. Trichothecene macrolides are valuable leading compounds, and many studies on their structure and anticancer activity have been carried out in recent years [11,12,13]. However, there were few reports concerning their anticancer mechanism [14,15], especially anticancer mechanism of 10,13-cyclotrichothecane derivatives, which were found mainly in symbiotic fungi. Mytoxin B (bearing a 12,13-epoxy group in the sesquiterpene residue) and myrothecine A (a 10,13-cyclotrichothecane derivative of mytoxin B) (Figure 1) are two trichothecene macrolides isolated from endophyte *Myrothecium roridum* IFB-E012 [1]. Our previous research had displayed that both mytoxin B and myrothecine A could inhibit the proliferation of the human hepatocarcinoma cell line SMMC-7721, which was closely related to the protein p27-mediated S phase cell cycle arrest in SMMC-7721 cells [16].

There are links between proliferation, cell cycle, and apoptosis. Besides cell cycle arrest, apoptosis (the type I programmed cell death, PCD) could also induce cell proliferation inhibition [17,18]. Apoptosis occurs in a wide variety of physiological conditions, with the role of removing harmful, damaged, or unwanted cells. Accumulating evidence has suggested that there is a balance between apoptosis and cell proliferation. Since apoptosis plays a critical role in cell survival, senescence, and homeostasis, apoptosis dysregulation is associated with a variety of diseases including cancer, autoimmune lymphoproliferative syndrome, AIDS, ischemia damage, and neurodegenerative diseases (such as Parkinson’s disease, Alzheimer’s disease, Huntington’s disease, and amyotrophic lateral sclerosis) [19]. Targeting apoptosis to develop new anticancer drugs has already become a new strategy. The plant-derived natural product gossypol (AT-101), that promotes cell apoptosis by inhibiting the activity of anti-apoptotic protein Bcl-2, had been in clinical trials for curing recurrent extensive stage small cell lung cancer [20,21].

Apoptosis is often an energy-dependent process which involves the activation of a group of cysteine proteases (i.e., caspases) [22]. Caspases (such as caspases-3, -8, and -9) play an important role in apoptosis [22] and usually regulates apoptosis by activating or deactivating substrate proteins. There are two major apoptosis pathways: the extrinsic death receptor pathway (caspase-8-dependent) and the intrinsic mitochondrial pathway (caspase-9-dependent). Both pathways finally activate caspase-3, which leads to irreversible apoptosis in cells [23]. The occurrence and development of tumors are closely related to abnormal cell signal transduction pathways. There are many tumor-related signaling pathways, such as MAPK pathway, PI3K/Akt/mTOR pathway, Wnt/β-Catenin pathway, IKK/NF-κB pathway, Notch pathway, and Hedgehog pathway. Among them, phosphoinositide 3-kinase (PI3K)/protein kinase B (PKB or Akt) signaling pathway is now regarded as one of the most critical regulators of a series of cell processes, including cell proliferation, apoptosis, and differentiation, and nutrient metabolism. Increasing evidence has shown that dysregulation of phosphatidylinositol 3-kinase (PI3K) signaling contributes to abnormal cell growth and cellular transformation in a variety of neoplasms. Since PI3K/Akt pathway is always overactivated in human tumor cells [24], a natural compound that could inhibit this pathway might be a potential anticancer drug or leading compound. Therefore, in the present study, we focused on the Bcl-2 protein family, caspases, and PI3K/Akt signaling pathway to investigate the induced apoptosis mechanism of mytoxin B and myrothecine A in SMMC-7721 cells.

## 2. Results and Discussion

### 2.1. Anti-Proliferation Effects of Mytoxin B and Myrothecine A against SMMC-7721 Cells

**Figure 2:**
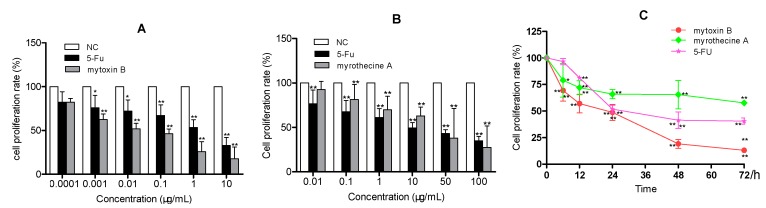
Anti-proliferation effects of mytoxin B and myrothecine A against SMMC-7721 cells. Cell viability was assessed using the MTT method. SMMC-7721 cells were treated with either control or the indicated concentrations of mytoxin B (**A**) and myrothecine A (**B**) for 24 h, respectively. SMMC-7721 cells were also treated with either 5-fluorouracil (1 μg/mL) or mytoxin B (0.1 μg/mL) and myrothecine A (10 μg/mL) for different times, respectively (**C**). Data were means ± SD of three independent experiments. Compared with negative control (NC), * *P* < 0.05, ** *P* < 0.01.

In our previous experiment, both mytoxin B and myrothecine A had showed an obvious anti-proliferation effect against SMMC-7721 cells at concentrations of 0.1~10 µg/mL [16]. In order to further investigate the effects of mytoxin B and myrothecine A on the proliferation of SMMC-7721 cells, MTT assay was performed to measure the viability of SMMC-7721 cells that were treated with preset concentrations of mytoxin B and myrothecine A for 24 h. 5-Fluorouracil (5-Fu) was co-assayed as a positive control. The result showed that the anti-proliferation activity of mytoxin B against SMMC-7721 cells was stronger than that of myrothecine A. Both mytoxin B and myrothecine A inhibited the growth of SMMC-7721 cells in a dose-dependent manner with IC_50_ values of 0.15 ± 0.04 and 25.5 ± 1.19 µg/mL, respectively, while the IC_50_ value of the positive control 5-Fu was 1.65 ± 0.05 µg/mL (Figure 2). The in vitro cytotoxicity of mytoxin B and myrothecine A also showed time-dependence, and effects of these two compounds to SMMC-7721 cells will be investigated after treatment for 24 h in the following studies (Figure 2).

### 2.2. Mytoxin B and Myrothecine A Induced Apoptosis in SMMC-7721 Cells

**Figure 3:**
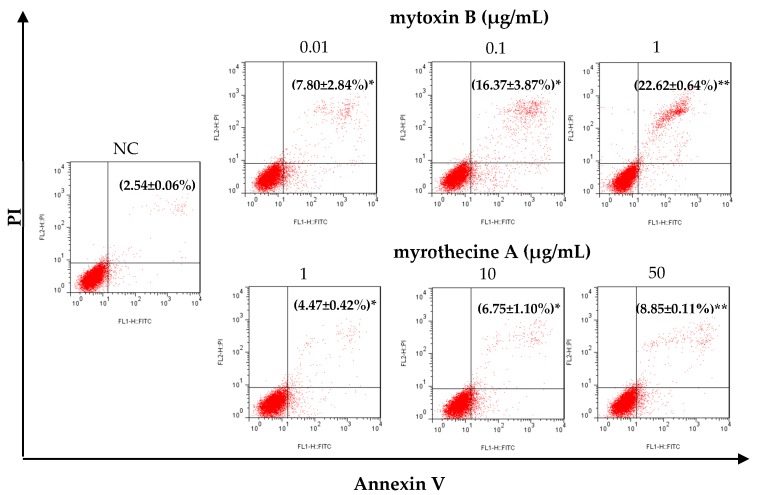
Mytoxin B and myrothecine A promoted apoptosis in SMMC-7721 cells. The apoptosis rates of SMMC-7721 cells treated with mytoxin B (0.01, 1, and 10 µg/mL) and myrothecine A (1, 10, and 50 µg/mL) for 24 h, respectively, were analyzed by flow cytometry using annexin V-FITC/PI staining. The apoptosis rate included early apoptosis and late apoptosis. Data were means ± SD of three independent experiments. Compared with negative control (NC), * *P* < 0.05, ** *P* < 0.01.

In order to find out whether the anti-proliferation effect of mytoxin B and myrothecine A against SMMC-7721 cells was associated with induced apoptosis, the apoptosis rate of SMMC-7721 cells after treatment with mytoxin B and myrothecine A for 24 h was examined using annexin V-FITC/PI dual stain assay. As shown in Figure 3, compared with negative control (with an apoptosis rate of 2.54 ± 0.06%, including early apoptosis and late apoptosis), apoptosis percentages of mytoxin B-treated SMMC-7721 cells increased to 7.80 ± 2.84% (*P* < 0.05), 16.37 ± 3.87% (*P* < 0.05), and 22.62 ± 0.64% (*P* < 0.01), respectively, and apoptosis percentages of myrothecine A-treated SMMC-7721 cells rose to 4.47 ± 0.42% (*P* < 0.05), 6.75 ± 1.10% (*P* < 0.05), and 8.85 ± 0.11% (*P* < 0.01), respectively. The result indicated that both mytoxin B and myrothecine A could induce SMMC-7721 cells apoptosis dose-dependently, however, apoptosis-inducing effect of mytoxin B was stronger than myrothecine A (Figure 3).

**Figure 4:**
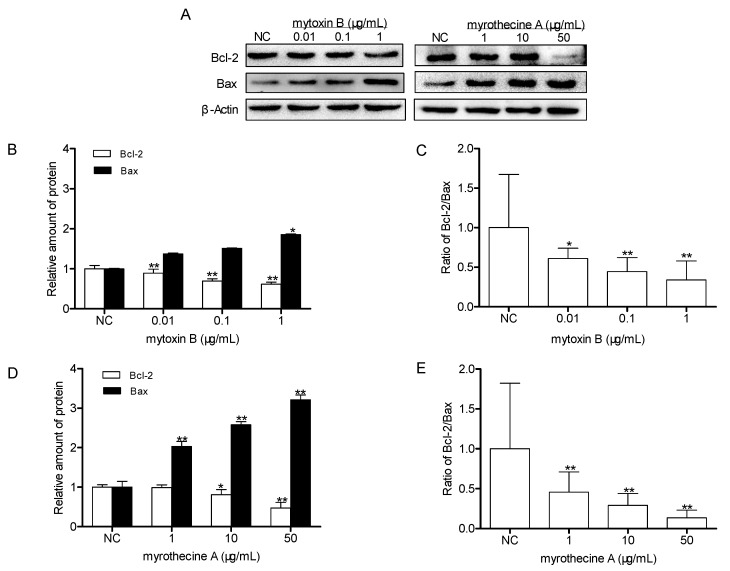
Effects of mytoxin B and myrothecine A on the expression levels of anti-apoptotic protein Bcl-2 and pro-apoptotic protein Bax in SMMC-7721 cells. Cells were treated with different concentration of mytoxin B (0.01, 1, and 10 µg/mL) and myrothecine A (1, 10, and 50 µg/mL) for 24 h, respectively. Cell lysates under various treatments were isolated and analyzed by Western blot analysis (**A**). Relative amount of Bcl-2 and Bax, and ratio of Bcl-2 and Bax in mytoxin B-treated cells (**B**,**C**). Relative amount of Bcl-2 and Bax, and ratio of Bcl-2 and Bax in myrothecine A-treated cells (**D**,**E**). The experiment was independently repeated three times. Compared with negative control (NC), * *P* < 0.05, ** *P* < 0.01.

The Bcl-2 (B cell lymphoma-2) protein family is the key regulator of apoptosis which regulates apoptosis by controlling mitochondrial permeability. In the present study, the expression levels of anti-apoptotic protein Bcl-2 and pro-apoptotic protein Bax were determined using Western blot analysis. As shown in Figure 4, after being cultured for 24 h in the presence of mytoxin B and myrothecine A, respectively, the decrease of Bcl-2 expression and the increase of Bax expression were observed in a dose-dependent manner in treated cells significantly, which meant that both mytoxin B and myrothecine A had a proapoptotic effect on SMMC-7721 cells. Bcl-2 protein family include a number of anti-apoptotic members (such as Bcl-2, Bcl-XL, Bcl-W, Bfl-1, and Mcl-1) and pro-apoptotic members (such as Bax, Bak, Bad, Bcl-XS, Bid, Bik, Bim, and Hrk) [25]. Among them, the *bcl*-2 proto-oncogene was the major gene to inhibit cell apoptosis. The anti-apoptotic protein Bcl-2 resides on the outer mitochondrial membrane and prevents apoptosis by inhibiting the activation of the pro-apoptotic members Bax and Bak [26]. Therefore, Bcl-2 down-expression is associated with Bax-mediated apoptosis. The balance of Bcl-2/Bax also plays an important role in the regulation of apoptosis [27]. The ratio of Bcl-2/Bax was reduced in both mytoxin B- and myrothecine A-treated cells in a dose-dependent manner, which might result in a reduction of dimerisation complex of Bcl-2 and Bax [26]. Bax translocated to the mitochondria to form a homodimer, promoting mitochondrial outer membrane permeabilization and the release of cytochrome c from the mitochondria into cytosol [28]. Then, caspase-9 was activated, which further activated caspase-3 and executed the apoptotic program through the mitochondrial pathway [23].

The loss of mitochondrial structural integrity and the permeabilization of mitochondrial membrane always lead to cell apoptosis, and oxidative stress plays an important role in the mitochondrial apoptosis pathway. Du et al. found that trichothecin (a simple trichothecene compound) promoted the production of reactive oxygen species (ROS) in HepG2 cells in a dose- and time-dependent manner, followed by the increment of Ca^2+^ concentration that might be due to the stimulation of ROS. Meanwhile, the mitochondrial membrane potential (ΔΨm) was decreased in a dose-dependent manner in trichothecin-treated HepG2 cells. They proposed that ROS may be a critical initiator of trichothecin-induced apoptosis in HepG2 cells [29]. Ye et al. also found that increasing of the mitochondrial membrane permeability and loss of the mitochondrial membrane potential enhanced the apoptosis rate significantly in HepG-2 cells after treatment with mytoxin B or epiroridin acid for 24 h [14].

### 2.3. Induced Apoptosis by Mytoxin B and Myrothecine A in SMMC-7721 Cells Involved in Both the Death Receptor Pathway and the Mitochondrial Pathway

**Figure 5:**
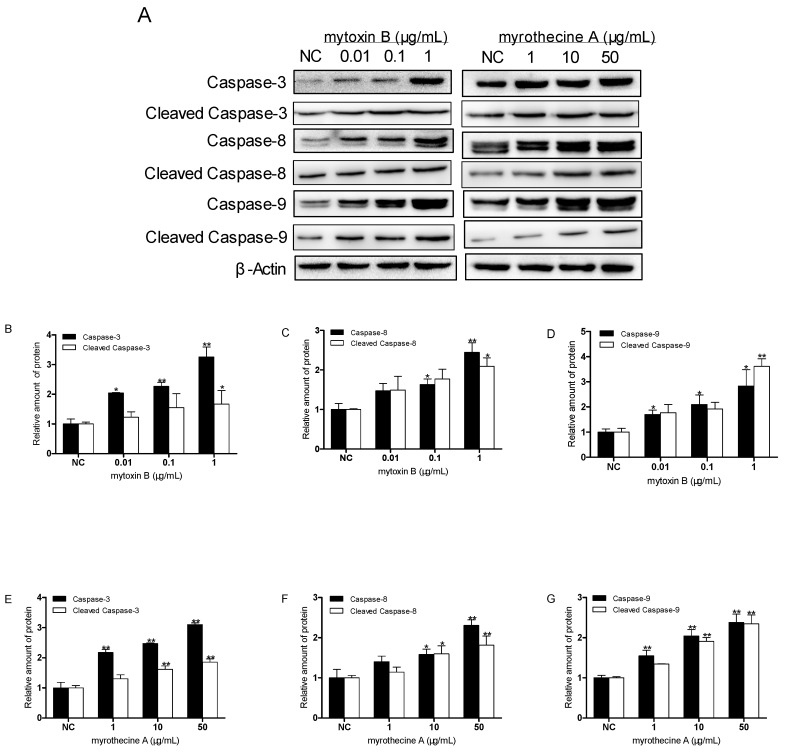
Effects of myrothecine A and mytoxin B on the expressions of caspases-3, -8, and -9, and cleaved caspases-3, -8, and -9 in SMMC-7721 cells. Cells were treated with different concentration of mytoxin B (0.01, 1, and 10 µg/mL) and myrothecine A (1, 10, and 50 µg/mL) for 24 h, respectively. Cell lysates under various treatments were isolated and analyzed by Western blot analysis (**A**). Relative amount of caspases-3, -8, and -9, and cleaved caspases-3, -8, and -9 in mytoxin B-treated cells (**B**–**D**). Relative amount of caspases-3, -8, and -9, and cleaved caspases-3, -8, and -9 in mytoxin A-treated cells (**E**–**G**). The experiment was independently repeated three times. Compared with negative control (NC), * *P* < 0.05, ** *P* < 0.01.

As a family of effector proteins in apoptosis, caspase activation can be regarded as a direct or indirect marker of apoptosis. In order to disclose the apoptotic pathway and the effect of caspases in induced apoptosis by mytoxin B and myrothecine A, SMMC-7721 cells were treated with these two compounds for 24 h, respectively, and the levels of caspases-3, -8, and -9, and cleaved caspases-3, -8, and -9 were examined by Western blot analysis. The result showed that the expression levels of caspases-3, -8, and -9, and cleaved caspases-3, -8, and -9 in treated SMMC-7721 cells were all upregulated in a dose-dependent manner (Figure 5), which demonstrated that mytoxin B and myrothecine A induced SMMC-7721 cell apoptosis via caspase activation.

Caspases are usually divided into upstream initiators and downstream effectors according to their structure and function during apoptosis. Effector caspases, such as caspase-3, contain only a short prodomain, whereas initiator caspases-8 and -9 have a long prodomain and exert regulatory roles by activating downstream effector caspases [30]. As an initiator caspase, caspase-9 is only involved in the mitochondrial pathway of apoptosis. Although caspase-8 could directly or indirectly activate two apoptosis pathways [31], it is still regarded as a marker protein in extrinsic death receptor pathway [22,32]. Caspases-8 and -9 finally activated caspase-3 that cleaved of a lot of substrates, including proteins involved in signal transduction, transcription, cell cycle control, and DNA replication and repair [33]. The activation of caspase-3 in apoptosis seems to be an irreversible step towards cell death [34]. Accordingly, the result also suggested that the apoptosis induced in SMMC-7721 cells by mytoxin B and myrothecine A was involved not only in the death receptor pathway but also the mitochondrial apoptosis pathway.

### 2.4. Involvement of PI3K/Akt Signaling Pathway in Mytoxin B- and Myrothecine A-Induced SMMC-7721 Cell Apoptosis

**Figure 6:**
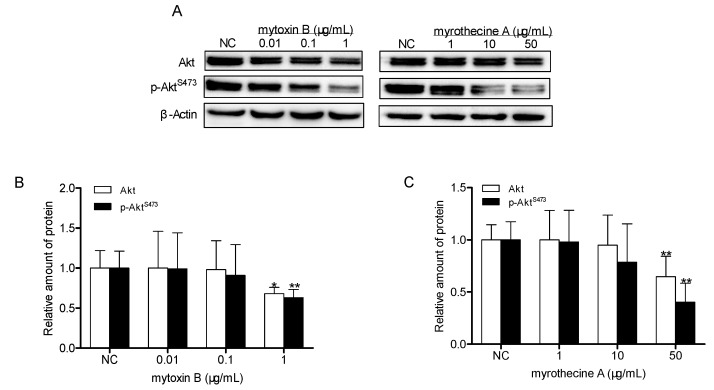
Effects of mytoxin B and myrothecine A on the expressions of Akt and phosphorylated Akt in SMMC-7721 cells. Cells were treated with different concentration of mytoxin B (0.01, 1, and 10 µg/mL) and myrothecine A (1, 10, and 50 µg/mL) for 24 h, respectively. Cell lysates under various treatments were isolated and analyzed by Western blot analysis (**A**). Relative amount of Akt and p-Akt^S473^ in mytoxin B-treated cells (**B**). Relative amount of Akt and p-Akt^S473^ in myrothecine A-treated cells (**C**). The experiment was independently repeated three times. Compared with negative control (NC), * *P* < 0.05, ** *P* < 0.01.

PI3K/Akt is an important signal transduction pathway mediated by tyrosine kinase receptors [35]. The PI3K/Akt signaling pathway acts as a sensor in response to extracellular stimuli and mediating the cellular signals, thereby playing a critical role in cell apoptosis and proliferation by affecting the activity of downstream effector molecules. In order to verify whether the PI3K/Akt pathway takes part in the anti-proliferation effects of mytoxin B and myrothecine A on SMMC-7721 cells, the expression and phosphorylation levels of Akt in treated SMMC-7721 cells were examined by Western blot analysis. The result showed that there were consistent decreases of Akt and p-Akt^S473^ in SMMC-7721 cells after 24 h treatment with mytoxin B and myrothecine A, respectively (Figure 6), indicating that the anti-proliferation effects of mytoxin B and myrothecine A against SMMC-7721 cells were related to deactivation of the PI3K/Akt pathway.

**Figure 7:**
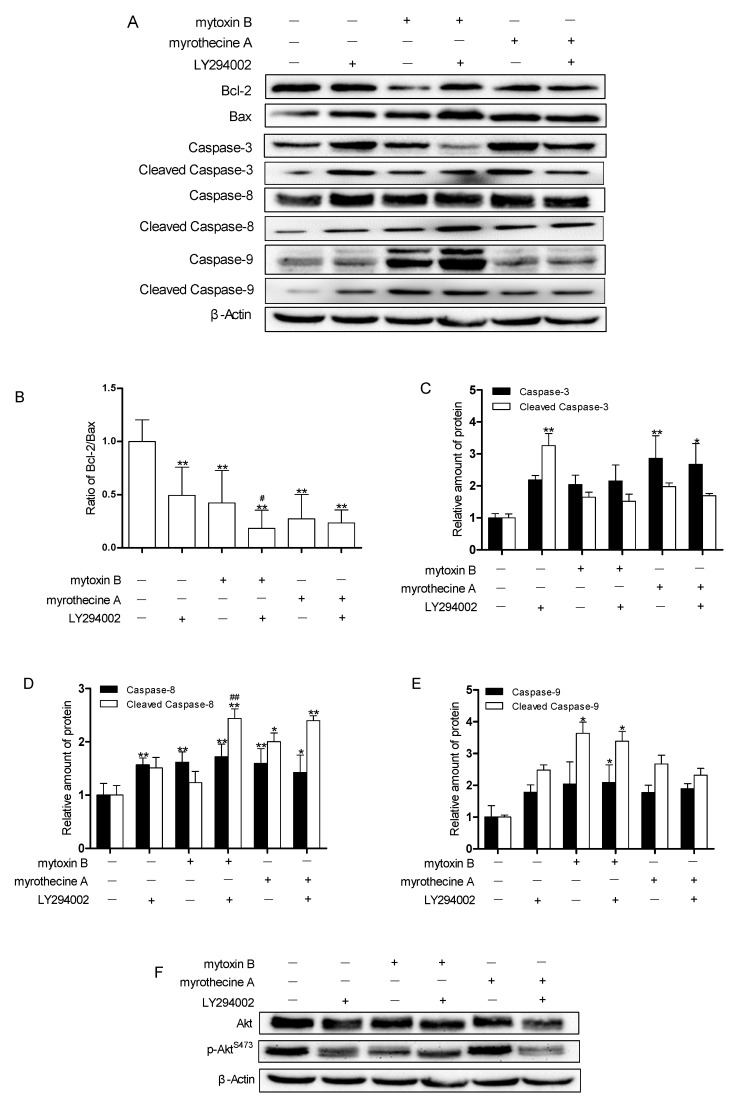

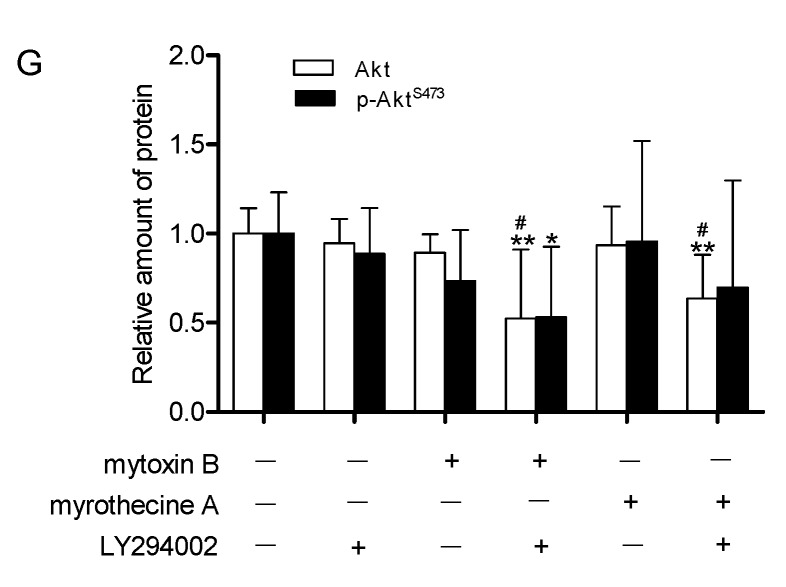
Effects of mytoxin B and myrothecine A on the PI3K/Akt signaling pathway and the role of PI3K/Akt pathway in mytoxin B- and myrothecine A-induced apoptosis in SMMC-7721 cells. Cells were treated with/without LY294002 for 30 min prior to exposure to mytoxin B and myrothecine A for 24 h, respectively. (I) Control; (II) LY294002 (20 μM); (III) mytoxin B (0.1 μg/mL); (IV) LY294002 (20 μM) + mytoxin B (0.1 μg/mL); (V) myrothecine A (10 μg/mL); (VI) LY294002 (20 μM) + myrothecine A (10 μg/mL). Cell lysates under various treatments were isolated and analyzed by Western blot analysis (**A**,**F**). Ratio of Bcl-2 and Bax in treated cells (**B**). Relative amount of caspase-3 and cleaved caspase-3 in treated cells (**C**). Relative amount of caspase-8 and cleaved caspase-8 in treated cells (**D**). Relative amount of caspase-9 and cleaved caspase-9 in treated cells (**E**). Relative amount of Akt and p-Akt^S473^ in treated cells (**G**). The experiment was independently repeated three times. Compared with negative control (NC), * *P* < 0.05, ** *P* < 0.01. Compared with single treatment group, ^#^ *P* < 0.05, ^##^ *P* < 0.01.

Further experiments were carried out to confirm whether the inhibition of PI3K/Akt signaling pathway by mytoxin B and myrothecine A could induce apoptosis in SMMC-7721 cells. LY294002 (a potent and specific PI3K inhibitor) was used as a control in this experiment. LY294002 has been reported to specifically inhibit the catalytic activity of PI3Kp110 subunit, block the activation of PI3k/Akt pathway, improve the apoptosis rate of cells, and effectively increase the sensitivity of chemotherapy drugs to cancer cells. In the present study, LY294002 treatment increased the expressions of caspase-8 and cleaved caspase-3, and decreased the ratio of Bcl-2/Bax, resulting in apoptosis in SMMC-7721 cells by inhibiting the PI3K/Akt signaling (Figure 7). Meanwhile mytoxin B treatment increased the expression of caspase-8 and cleaved caspase-9, and decreased the ratio of Bcl-2/Bax, and myrothecine A treatment increased the expression of caspases-3 and -8, and cleaved caspase-8, and decreased the ratio of Bcl-2/Bax. Considering the results shown in Figure 5 and Figure 6, it could be deduced that the effects of mytoxin B and myrothecine A were very similar to LY294002, and both could also deactivate Akt and p-Akt^S473^ in a PI3K-dependent manner. In addition, compared with mytoxin B or myrothecine A alone, the treatment in combination with LY294002 obviously evoked the downregulation of Akt (Figure 7), which meant that inhibition of the PI3K/Akt pathway strengthened mytoxin B- or myrothecine-triggered apoptosis in SMMC-7721 cells to some extent. The above findings support the idea that both mytoxin B and myrothecine A could reduce SMMC-7721 cell survival through induced apoptosis by inhibiting the PI3K signaling pathway (Figure 7). However, the obviously increased level of cleaved caspase-8 expression and the decreased ratio of Bcl-2/Bax were observed in response to the combination treatment of mytoxin B with LY294002 (Figure 7), indicating that LY294002 has a different influence on mytoxin B and myrothecine A.

**Figure 8:**
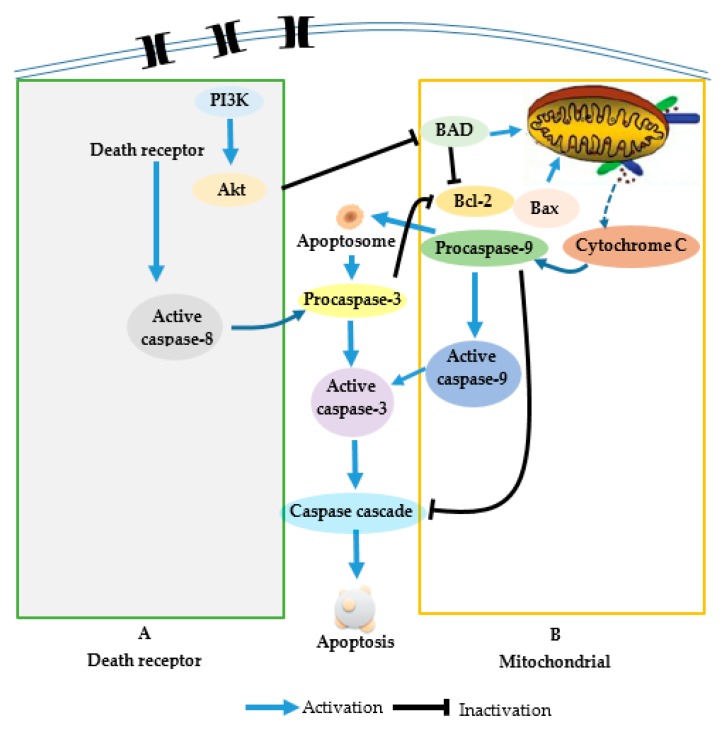
Schematic representation of apoptotic events.

PI3K/Akt signaling pathway could inhibit apoptosis through various mechanisms [36,37]. The activation of PI3K/Akt pathway provides cells with a survival signal that allows them to withstand apoptotic stimuli. Aberrant activation of the PI3K/Akt pathway has been associated with multiple human cancers, including liver cancer, breast cancer, and thyroid cancer. Akt, the downstream effector of PI3K, is activated by site-specific phosphorylation at Thr308 or Ser473. Akt takes center stage in angiogenesis signaling [38] and Akt activation is closely related to tumorigenesis. Through phosphoinositol-dependent kinases, Akt is phosphorylated and activated to further phosphorylate other downstream protein substrates, thus leading to various signaling cascades that affect cellular functions. PI3K/Akt pathway has become a therapeutic target for cancer treatment. Some drugs targeting this pathway have already been through clinical trials [39]. Therefore, mytoxin B and myrothecine A might be two potential anticancer lead compounds targeting the PI3K/Akt pathway (Figure 8).

Several studies have reported that the 12′,13′-epoxide group was critical to cytotoxicity of trichothecene macrolides. In the present study, mytoxin B inhibited the proliferation of SMMC-7721 cells with an IC_50_ value of 0.15 ± 0.04 µg/mL, while myrothecine A showed an IC_50_ value of 25.5 ± 1.19 µg/mL to SMMC-7721 cells. However, there is no other obvious mechanism difference between these two compounds in the following experiment. Thus, the anticancer mechanism of mytoxin B and myrothecine A against SMMC-7721 cells needs further investigation.

There are several dysregulation cell signaling pathways in tumor cells. The MAPK signaling pathway is another important intracellular signal transduction pathway which can transmit the extracellular stimuli to cells and even to the nucleus, inducing cell biological responses such as proliferation, differentiation, transformation, and apoptosis. Gratton J.-P. et al. [40] had found that there is crosstalk between the PI3K/Akt and p38 MAPK pathways, so it may be worthwhile to explore the interaction between these two tumor-related pathways in apoptosis induced in SMMC-7721 cells by mytoxin B and myrothecine A. Autophagy, the type II programmed cell death, also plays an important role in tumorigenesis, metastasis, and therapy [25]. Autophagy has shown a close relation with apoptosis [19] and might exert its influence in mytoxin B- and myrothecine A-induced apoptosis. The abovementioned research strategies might lead to discovery of the effect of the 12′,13′-epoxide group in the cytotoxicity of trichothecene macrolides.

## 3. Materials and Methods

### 3.1. General Experimental Procedures

Cells were incubated using a Thermo Forma CO_2_ incubator (Thermo Fisher Scientific, Waltham, MA, USA). The absorbance of 96-well plates was measured on an ELX800UV microplate reader (Bio-Tek, Winooski, VT, USA). Percentages of apoptotic cells were recorded using BD FACSCalibur Flow Cytometry (Becton Dickinson and Company, Franklin Lakes, NJ, USA). Western blot analysis was conducted using a JY-ZY5 SDS-PAGE system (Bio-Rad Laboratories, Inc., Hercules, CA, USA) and a CB FluorChemE automatic gel imaging analysis system (Santa Clara, CA, USA).

The human hepatocellular carcinoma cell line SMMC-7721 was provided by the American Type Culture Collection, Rockefeller, MD, USA. Mytoxin B and myrothecine A were isolated from endophyte *Myrothecium roridum* IFB-E012 in our previous research [1]. RPMI 1640 medium, fetal bovine serum, and 0.25% trypsase were purchased from Gibco Co., Grand Island, NY, USA. 3-(4,5-Dimethyl-2-thiazolyl)-2,5-diphenyl-2-H-tetrazolium bromide (MTT) was purchased from Sigma-Aldrich Co., LLC., St. Louis, MO, USA. The Annexin V-FITC apoptosis detection kit was a product of Becton, Dickinson and Company, Franklin Lakes, NJ, USA. The whole protein extraction kit was supplied by Beijing Solarbio Science & Technology Co., Ltd., Beijing, China. A penicillin–streptomycin mixed solution was purchased from Hyclone Laboratories Inc., Logan, UT, USA. Propidium iodide (PI) and the BCA Protein Quantitation Kit were bought from Jiangsu Keygen Biotech Co., Ltd., Nanjing, China. Protein marker was produced by Fermentas Inc., Burlington, ON, Canada. The SDS-PAGE Gel Preparation Kit was purchased from Shanghai Beyotime Biotechnology, Shanghai, China. LY294002 was a product of MedChemexpress, Monmouth Junction, NJ, USA. Super ECL Plus ultra-sensitive liquid was supplied by Beijing Applygen Technologies Inc., China. Monoclonal antibodies against human caspases-3, -8, and -9, Akt, phospho-Akt^S473^ (p-Akt^S473^), Bcl-2, Bax and β-actin were all purchased from Cell Signaling Technology, Inc., Boston, MA, USA. Horseradish peroxidase-labeled anti-mouse and anti-rabbit IgG secondary antibodies were purchased from Wuhan Boster Biological Technology, Ltd., China and Cell Signaling Technology, Inc., Boston, MA, USA, respectively. 5-Fluorouracil (5-Fu) was produced by Tianjin Kingyork Pharmaceutical Co., Ltd., Tianjin, China. The 96-well plate was supplied by Corning Inc., Corning, NY, USA. The PVDF membrane was bought from Millipore Corp., Billerica, MA, USA.

### 3.2. Cell Proliferation Assay

The MTT method was used to measure the cell viability. SMMC-7721 cells in exponential growth phase were seeded in 96-well plate at a density of 1 × 10^4^ cells/well in 100 µL RPMI 1640 medium, and the subsequent incubation was permitted at 37 °C with 5% CO_2_ for 24 h before assessment. Mytoxin B and myrothecine A at preset concentrations were added to five replicate wells and treated cells for 24 h, respectively, with 5-fluorouracil (5-Fu) as a positive reference. MTT (10 µL) was added to each well and the plate was continued to culture for 4 h. Then, the supernatant of each well was removed and 150 µL DMSO was added to dissolve the formazan. The absorbance at 490 nm of each well was measured using an automatic ELISA plate reader. The experiment was repeated in triplicate. Cell viability was expressed as percentage of proliferation cells in comparison with the negative control.

### 3.3. Annexin V-FITC/PI Dual Staining Assay

The apoptosis rate of SMMC-7721 cells was detected through a annexin V-FITC/PI dual staining assay. SMMC-7721 cells in exponential growth phase was seeded in 6-well plate at 1 × 10^6^ cells/well, and were treated with mytoxin B and myrothecine A at different concentrations. After incubation for 24 h, cells were washed with cold PBS and resuspended in 100 µL annexin V/PI binding buffer, then incubated with 5 µL of annexin V and 1 µL of PI for 15 min at room temperature in the dark. Viable cells were scored as those that were negative for annexin V and PI. The stained cells were analyzed by flow cytometry to determine the percentages of apoptosis cells, including annexin V^+^/PI^−^ (early apoptosis) and annexin V^+^/PI^+^ (late apoptosis) cells.

### 3.4. Western Blot Analysis

SMMC-7721 cells were treated with mytoxin B and myrothecine A for 24 h, respectively, and protein extraction was performed following the whole protein extraction kit protocol. After quantification according to BCA protein kit instruction, equal loads of proteins were resolved by SDS-PAGE and transferred onto PVDF membranes. The membranes were blocked in 5% nonfat dry milk in TBST (TBS-0.05% Tween-20) with gentle shaking 2 h, and incubated with primary antibody overnight at 4 °C. After being washed with TBST three times, the membranes were incubated with the appropriate HRP-conjugated secondary antibody and then detected using the Super ECL Plus ultra-sensitive liquid. Images were collected and the respective bands were quantitated by densitometric analysis using the Image J program (National Institutes of Health, Bethesda, MD, USA).

### 3.5. Statistical Analysis

Data were expressed as means ± SD. IC_50_ value was calculated through an improved Karber’s method. Figures were obtained using GraphPad 5.0 and the difference between two groups was analyzed by one-way anova in SPSS 20.0. *P* < 0.05 indicated a significant difference, while *P* < 0.01 indicated a very significant difference.

## 4. Conclusions

Mytoxin B and myrothecine A are two satratoxin-type trichothecene macrolides. Mytoxin B contains a 12,13-epoxy group in the sesquiterpene residue, while myrothecine A forms a 10,13-carbocycle through acid rearrangement of the 12,13-epoxy group. Early studies discovered that the 12,13-epoxy group in the sesquiterpene residue was critical to the cytotoxicity of trichothecene macrolides [41], however, the structural difference between mytoxin B and myrothecine A did not lead to different in vitro anticancer mechanisms against SMMC-7721 cells in the present study. Both mytoxin B and myrothecine A could induce apoptosis in SMMC-7721 cells via the PI3K/Akt signaling pathway and the induced apoptosis was involved in not only the death receptor pathway but also the mitochondrial apoptotic pathway. The abovementioned results provided new clues for trichothecene toxins in developing anticancer drugs, and differences in the anticancer mechanisms between mytoxin B and myrothecine A could be disclosed through further research.
